# The Massachusetts Medical Society

**Published:** 1871-01

**Authors:** 


### THE MASSACHUSETTS MEDICAL SOCIETY—ITS LATE ACTION.

At the last meeting of the American Medical Association, Drs. H. R. Storer and J. S. Sullivan presented a protest from the Boston Gynæological Society, against the admission of the delegates from the Massachusetts Medical Society, upon the ground that the latter Society held within its membership irregular practitioners. Dr. A. Stille, Chairman of the Committee on Ethics, reported (which we abridge,) "that the charge of tolerating in the Massachusetts Medical Society, men acknowledged to have been Homœpaths and Eclectics, is fully proven, and is plainly in violation of the Code of Ethics." But the parties making the charge being members of that Society, having failed to make any specific charge against them in the Massachusetts Medical Society, the Committee thought such steps should have been taken before appealing to the Association, and they, therefore, recommended "that the Committee of Registration should register all regularly accredited delegates from that Society, to the present meeting." The Committee further recommend "that unless said Society takes the necessary steps to purge itself of irregular practitioners, it ought not to be entitled to future representation" in the Association.

At the annual meeting of the Massachusetts Medical Society, in response to the demands of the American Medical Association, the following resolution was adopted:

"*Resolved*, That the Massachusetts Medical Society hereby expel from fellowship all those who publicly profess to practice in accordance with any exclusive dogma, whether calling themselves homœpaths, hydropaths, eclectics or what not, in violation of the code of Ethics of the American Medical Association."

The action of Drs. Storer and Sullivan, in reporting the irregularities of the Massachusetts Medical Society, cannot be justly censured by the profession, viewing the matter from an ethical stand-point. Prior to the last meeting of the American Medical Association, no action had been taken requiring State Societies or Associations to dispose of all cases of violations of the Ethics before submitting them to the higher body. It now, however, occupies the position of a high Court of Appeal, upon all questions in Ethics, that may hereafter arise in State and local Societies.

In order to enable our readers to determine for themselves the justice of the action of the American Medical Association, and that of the Massachusetts Medical Society, as well as the importance of the questions involved, we submit a few interrogations:

1st. When the Massachusetts Medical Society was first organized, was it not necessary, in order to its recognition, by the American Medical Association, that its Constitution and Code of Ethics, should harmonize with those of that Association? Had it been otherwise, could the Society have been admitted to representation in the Association? Certainly not.

2d. If, when applying for representation in the Association, it had been announced or discovered, that it (the Massachusetts

Medical Society,) contained within its membership irregular practitioners—or parties under professional condemnation and excommunication from another Society or organization—would its delegates have been entitled to, or allowed, representation, in the Association? Of course not.

3d. Could the Massachusetts Medical Society have taken any other action than the one recommended by the American Medical Association, (i. e. to have "purged out" the offending members from its body,) without violating the Code of Ethics, and bringing reproach upon the professional integrity of its members? Surely not.

4th. Can the American Medical Association in future, receive delegates from the Massachusetts Medical Society, unless that Society carry into *practical* operation the resolution it adopted at its last annual meeting? We think not.

5th. Suppose the expelled members from the Massachusetts Medical Society, while under the ban of excommunication, should organize a new Society, pretending to do so under the Constitution and Code of Ethics of the American Medical Association, would such Society or its members be entitled to recognition by, or representation in, that Association? Assuredly not.

6th. Is it possible (ethically, of course,) for regular practitioners to unite with irregular, or expelled members, from any regularly organized Society or Association, in organizing a new Society, without contamination, and without subjecting themselves to the charge of irregularity, by association and affiliation? Unquestionably not.

